# Conversion rate to open surgery during transanal total mesorectal excision (TaTME) for rectal cancer: a single-center experience

**DOI:** 10.1007/s13304-024-01844-0

**Published:** 2024-04-28

**Authors:** Flavio Tirelli, Laura Lorenzon, Alberto Biondi, Ilaria Neri, Gloria Santoro, Roberto Persiani

**Affiliations:** 1 General Surgery Unit, Fondazione Policlinico Universitario Agostino Gemelli IRCCS, Rome, Italy; 2 Catholic University, Largo Francesco Vito 1, 00168 Rome, Italy

**Keywords:** rectal cancer, minimally-invasive surgery, transanal total mesorectal excision, conversion

## Abstract

Minimally invasive techniques for rectal cancer have demonstrated considerable advantages in terms of faster recovery and less post-operative complications. However, due to the complex anatomy and a limited surgical field, conversion to open surgery is still sometimes required, with a negative impact on the short-and long-term outcomes. The purpose of this study was to analyse the conversion rate to open abdominal surgery during laparoscopic transanal total mesorectal excision (TaTME) procedures performed at a high-volume Italian referral center. All consecutive TaTME performed for mid-to-low rectal cancer between 2015 and 2023 were reviewed, independently if treated with a primary anastomosis (with/without a diverting ostomy) or an end stoma. All procedures were performed using a standardized approach by the same surgical team. Patients with benign diagnosis that underwent different-from rectal resection procedures and cases pre-operatively scheduled for open surgery were excluded. The primary outcome of interest was the rate of conversion, defined as an un-planned intraoperative switch to open surgery using a midline laparotomy. Secondary aims included the comparison of patients who had a longer vs shorter operative time. Out of 220 patients, 210 were selected. In 187 cases, a primary anastomosis was performed, while 23 patients received a terminal colostomy (1 in the converted group; 22 in the full MIS- TaTME group, 10.6%). A surgical approach modification occurred in two cases, with a conversion rate of 0.95%. Median operative time was 281 min. Reasons for conversions included intra-operative difficulties impairing the mini-invasive procedure without intra-operative complications in one case, and difficulties in the laparoscopic control of an intraoperative bleeding due to a splenic lesion in another patient. Male sex and a higher BMI were found to be statistically significantly associated to longer operative time (respectively: *p* = 0.001 and *p* = 0.0025). In a high-volume center, a standardized TaTME is associated to a low conversion rate to open abdominal surgery.

## Introduction

New and advanced mini-invasive techniques for rectal cancer treatment offer considerable advantages in terms of shorter patient’s hospitalization, less postoperative pain, reduced rate of surgical site infections, and better aesthetic results [1].

Nonetheless, challenging intra-operative findings or issues may result in conversion to open surgery even for experienced surgeons.

According to the 2013 Delphi consensus, conversion is defined as an intraoperative switch from either a robotic or laparoscopic approach to an open abdominal approach because of anticipated operative difficulty or logistic considerations (preemptive conversion) or because of a complication or operative difficulty after a considerable amount of dissection (reactive conversion) [2, 3].

The impaired visibility in a narrow pelvis, the limited instrument motion, the poor ergonomics and the unavoidable dependency on assistants for retraction and camera handling can indeed affect the possibility to achieve complete laparoscopic surgeries [4, 5].

Novel approaches, such as the robotic and the transanal total mesorectal excision (TaTME), have emerged in attempting to overcome the technical limitations of full trans-abdominal laparoscopic procedures.

The robotic technique provides the considerable advantage of 3D vision and articulating wristed instruments that result in superior ergonomics and the possibility of a more accurate pelvic dissection [6].

The alternative “bottom-up” TaTME approach was thought to provide a better accessibility to low or bulky tumours, especially in case of narrow, male pelvis or in obese patients, offering the possibility of a safe and complete dissection of the mesorectal fascia. Although not free from complications, this technique allows the realization of very low anastomosis in patients that would not have otherwise undergo a restorative or sphincter-preserving procedure [7, 8]. Despite available evidence reporting the transanal approach to be associated with a reduced conversion rate to open surgery [5, 9, 10], recent systematic reviews did not detect significant differences when comparing to laparoscopic or robotic techniques [7, 11–13].

However, past series analysing outcomes of laparoscopy in rectal resections, reported a rate of incisional hernias of 6%, and this outcome was related to conversion [14]. Such events increase health-related costs, but literature, to-day, focused more on small series [5], national datasets [9], or pooled analysis of data [5, 7, 9–13], than on the competitive advantage of this approach in a single high-volume center.

The primary aim of this study was thus to analyse the conversion rate from laparoscopic to open abdominal surgery during TaTME procedures in a high-volume Italian center. The secondary aim was to describe the study group’s restorative rate and the conversion rate in the restorative surgeries subgroup.

## Methods

All consecutive patients who underwent TaTME for low and mid rectal cancers (respectively up to 6 cm and 7–11 cm from the anorectal junction) at the Fondazione Policlinico Universitario A. Gemelli in Rome from May 2015 to May 2023 were collected in a prospectively maintained database and reviewed for the purpose of the analysis. The surgical technique has been standardized since its adoption, and it consists of a combined and double-equipe synchronous trans-anal/laparoscopic trans-abdominal procedure (Cecil approach) [14].

Patients were included independently if treated with a primary colorectal or coloanal anastomosis (with or without loop ileostomy/colostomy) or with a terminal colostomy (Hartman’s and Miles procedures). Patients with benign diagnosis (e.g., inflammatory bowel disease) that underwent total or subtotal colectomy, proctocolectomy were also excluded.

Patients preoperatively scheduled for a laparotomic transabdominal resection were recorded but excluded from the current analysis. Clinical (sex, BMI, previous abdominal surgery), cancer-related (clinical stage) and operative features (anastomosis fashioning vs terminal ostomy, conversion to laparotomic transabdominal approach) were recorded.

Conversion to laparotomy was defined as an un-planned intraoperative switch from a laparoscopic to an open abdominal approach because of anticipated operative difficulty or logistic considerations (strategic conversion) or because of a complication (reactive conversion), requiring a midline laparotomy. The study objective was the conversion rate to laparotomic transabdominal approach. Secondary outcomes were the restorative rate following mini-invasive surgery, the conversion rate in mini-invasive restorative surgery and a comparison of patients with a longer vs shorter operative time.

The protocol has been notified and approved by the Institutional Review Board (Study ID:6496, Protocol number 0006070).

*Statistics*. Categorical variables were reported using frequencies and percentages; continuous variables were reported using median, mean values and standard deviation (SD) and inter-quartile ranges (IQR). A descriptive sub-analysis of converted patients was also performed. Patients were divided in two groups (below and above median operative time) and a univariate analysis using *t*-test and χ^2^ test was performed to assess a possible correlation of demographics, clinical and oncological features with operative time.

## Results

Of the 220 patients who underwent TaTME during the study period, 6 patients were excluded because they underwent total or subtotal colectomy for benign diagnosis and 4 ones because of preoperatively scheduled transabdominal laparotomic approach. In two cases, the choice of open technique was related to the presence of previous laparotomic accesses: the patients had peri-anastomotic recurrent disease and were previously treated with laparotomic anterior rectal resection. In the other two cases, combined liver surgery for synchronous hepatic metastasis resulted in since-the-beginning laparotomic abdominal approach. Two hundred ten patients were eligible for enrolment. A modification of the surgical approach occurred in two cases, with a conversion rate of 0.95%.

In one case of mid rectal lesion, it was a reactive conversion: a lower pole splenic lesion occurred during the splenic flexure mobilization due to the presence of dense splenic-omental adherences. The bleeding was difficult to laparoscopically control. It was, therefore, necessary to convert to the laparotomic approach and perform a splenectomy. TaTME was then completed, and a mechanical colorectal anastomosis was realised. In the other case of low rectal tumour, it was a pre-emptive conversion. The patient underwent diagnostic laparoscopy that confirmed the pre-operative TC findings of omental and ileal mesentery nodules. The lesions were biopsied, and an extemporaneous histological examination was performed that confirmed the metastatic nature of the nodules. The transabdominal equipe started the laparoscopic dissection but, for anticipated operative difficulties, the procedure was completed though a laparotomic approach. In order to achieve oncological radicality, the patient underwent debulking (omentectomy, anterior parietal and bilateral diaphragmatic peritonectomy, anterior rectal resection with right seminal vesicle removal) with Hyperthermic Intraperitoneal Chemotherapy. In line with the preoperative strategy, the patient received TaTME with terminal colostomy. Thus, if we limit the analysis to patients undergoing restorative surgery, conversion rate was 1.1%. Of note, both of converted patients were males, with a conversion rate of 1.5% and 0%, in male and female population, respectively.

Among patients who underwent a full mini-invasive—not converted—TaTME (208 patients, 99%), 62.9% were male; the mean BMI was 25.09 ± 3 SD; 41.4% (87 patients) underwent previous abdominal surgery; in 64.3% of cases, the neoplastic lesion was clinically staged > T3 or N + (Table 1). In 187 cases, a primary anastomosis was performed, while 23 patients received a terminal colostomy (1 in the converted group; 22 in the mini-invasive group-10.6%) with a mini-invasive surgery restorative rate restorative rate of 89.4%. Among patients who underwent minimally invasive surgery with primary anastomosis, 60.7% were male, the mean BMI was 25.03 ± 8.9 SD, 42.4% underwent previous abdominal surgeries, 63.4% were clinically staged > T3 or N + . Among the subgroups of patients who underwent a mini-invasive non-restorative procedure, 86.3% were male; the mean BMI was 24.5 ± 4.3; 36.3% underwent previous abdominal surgery; 77.2% had a rectal tumour staged > T3 or N + (Table 1).

**Table 1:** Demographics, clinical and oncological features of patients who underwent full mini-invasive surgery with primary anastomosis and non-restorative mini-invasive surgery

	Mini-invasive surgery with primary anastomosis	Mini-invasive non-restorative surgery
Total *n* (%)	208 (99%)	22 (10.6%)
Male *n* (%)	131 (62.9%)	19 (86.3%)
Mean BMI	25.09 ± 3	24.5 ± 4.3
Previous abdominal surgeries *n* (%)	87 (41.4%)	8 (36.3%)
c ≥ T3 or *N* + *n* (%)	134 (64.3%)	17 (77.2%)

Median operative time was of 281 min, IQR 248–320. In patients who underwent a full mini-invasive TaTME procedure (208 patients), the univariate analysis was performed to assess the association of sex, age, BMI, ASA score, Charlson index, tumour’s distance from the anorectal junction, neoadjuvant therapy and tumour’s dimension (cT) with operative time. This analysis showed that male sex and higher BMI were associated to longer operative time and the correlation was of statistical value (*p* = 0.001; *p* = 0.0025; Table 2).

**Table 2:** Univariate analysis comparing patients according to the operative time in patients treated with full mini-invasive TaTME

	Below median operative time *n* patients: 107	Above median operative *n* patients: 101	*p* value
Sex			
Male	55 (51.4%)	76 (75.2%)	0.001*
Female	52 (48.6%)	25 (24.8%)	
Age (years)			
Mean (SD)	68.3 (12.1)	67.7 (10.4)	0.687
BMI			
Mean (SD)	24.6 (3.4)	25.8 (4.4)	0.025*
Charlson index			
Mean (SD)	2.1 (2.2)	2.5 (2.4)	0.208
NAD			
No	31 (29.0%)	30 (29.7%)	1
Yes	76 (71.0%)	71 (70.3%)	
ARJ distance (mm)			
Mean (SD)	60.7 (27.6)	60.1 (25.5)	0.852
ASA score			
ASA1	9 (8.4%)	5 (5.0%)	0.547
ASA2	77 (72.0%)	78 (77.2%)	
ASA3	21 (19.6%)	18 (17.8%)	
cT			
cT0-2	17 (16.8%)	22 (25.6%)	0.198
cT3-4	84 (83.2%)	64 (74.4%)	

*NAD* neoadjuvant therapy, *ARJ* anorectal junction

*statistical significance *p* < 0.05

## Discussion

The present study investigated the conversion rate to open abdominal surgery during combined (laparoscopic transabdominal/transanal) procedures for the treatment of mid- and low rectal cancer. Although this is not a cost-effectiveness analysis, the aim of this research was to emphasize the competitive advantage of the combined approach in reducing the conversion rate, even in technically complex cases, and to highlight the related benefit in terms of post-operative morbidity and outcomes. Indeed, conversion and the related laparotomy, is associated with a delay in post-operative functional recovery, a prolonged hospitalization and an increased risk of incisional hernias, which could require a further surgical procedure [14].

In this series, the reported rate has shown to be low and in line with available evidences from literature. It also presented a considerable restorative rate following TaTME, supporting the technical advantages offered by this mini-invasive approach, when performed in a high-volume center and after an accurate patient’s selection.

According to literature, conversion from laparoscopic to open surgery is reported ranging between 8 and 34% of cases, depending on different centres series, surgeons’ expertise and learning curve, demographic and oncological features of the study populations [12]. Conversion rate during TaTME varies from 1 to 1.9% [10]. In the present series, the conversion rate stands at 0.95%, thus in keeping with surgical series reported in literature so far.

In the study group, all the procedures were performed by the same surgical team. We may speculate that this could have improved surgeons’ expertise and positively affected the results. The transanal and transabdominal teams were both lead by two senior surgeons with a wide experience in mini-invasive colorectal surgery [15]. Both of the conversion-to-laparotomic-approach events occurred after the previously analysed learning phase [16]; however, it should be noticed that, in our previous analysis, learning curves were focused on the reduction on other adverse events, such as major complications, the anastomotic leakage or failure and the reoperation rate [16]. The optimization of the surgical technique and the team’s setup may have led nevertheless to a better patients' selection.

Conversion to open surgery is associated to worse post-operative outcomes, particularly in terms of overall morbidity rate and severe complications [17, 18]. Aside from the above-mentioned aspects, conversion itself has shown an individual negative impact on mid- and long-term oncological outcomes in rectal surgery [17, 19]. Different studies reported an increased R1 rate[18], a decreased overall survival and disease free survival rates [17, 19, 20] in patients who underwent conversion to laparotomic approach during mini-invasive procedures, even in case of matched study populations for T-stage, demographics and clinical features [21, 22].

Regardless the adopted mini-invasive approach in rectal surgery, male sex, advanced tumour stage and previous abdominal surgery were identified as documented risk factors for conversion to open surgery [4], and increasing BMI showed an incremental impact on surgical complexity¸ including conversion rate [23].Nonetheless, novel mini-invasive approaches represent one of the available solutions to mitigate the negative effect of overweight, with lower rates of poor postoperative outcomes in obese cohorts, thanks to the possibility of improved visualization during pelvic dissection, even in case of fatty, thick, and fragile tissues [23]. Operative time may be considered an indicator of technical difficulty and a parameter to identify more demanding procedures that have a higher risk of conversion, even if completed through a mini-invasive approach. In this context, male sex and increasing BMI disclosed a correlation to longer operative time in the present series. We reported a consistent percentage of patients with risk factors for conversion (male sex, overweight, previous abdominal surgery, advanced tumour stage) that underwent complete mini-invasive TaTME. We may speculate this positive result can support the advantages the transanal approach offers to overcome challenging situations that could have otherwise led to conversion. An important point to be considered is the centre volume and expertise. As previously reported, it represents a crucial factor that correlates to lower complications and reintervention rates, suggesting that minimally invasive techniques advantages require extensive experience and training to be really appreciated [11, 24, 25].

Nonetheless, results from this study need to be interpretated considering some limitations. Its retrospective design with lack of randomization, as well as the single-center nature with geographically restricted inclusion of patients precludes external validity.

## Conclusions

When performed in high-volume centers with a standardized procedure, after a proper learning curve and an accurate patients’ selection, minimally invasive surgery is beneficial and feasible, even in high-risk-for-conversion patients. The transanal approach represents an additional crucial advantage for the low conversion rate to open abdominal surgery in TaTME.

## Data Availability

Research data supporting this publication are available from the corresponding author on reasonable request.
